# DCBAN: A Dynamic Confidence Bayesian Adaptive Network for Reconstructing Visual Images from fMRI Signals

**DOI:** 10.3390/brainsci15111166

**Published:** 2025-10-29

**Authors:** Wenju Wang, Yuyang Cai, Renwei Zhang, Jiaqi Li, Zinuo Ye, Zhen Wang

**Affiliations:** College of Publishing, University of Shanghai for Science and Technology, Shanghai 200093, China; 233352874@st.usst.edu.cn (Y.C.); 233352879@st.usst.edu.cn (R.Z.); 233352883@st.usst.edu.cn (J.L.); 233352877@st.usst.edu.cn (Z.Y.); 233352871@st.usst.edu.cn (Z.W.)

**Keywords:** fMRI brain decoding, visual image reconstruction, deep learning, dynamic confidence Bayesian adaptive network, neuroimaging

## Abstract

**Background:** Current fMRI (functional magnetic resonance imaging)-driven brain information decoding for visual image reconstruction techniques faces issues such as poor structural fidelity, inadequate model generalization, and unnatural visual image reconstruction in complex scenarios. **Methods**: To address these challenges, this study proposes a Dynamic Confidence Bayesian Adaptive Network (DCBAN). In this network model, deep nested Singular Value Decomposition is introduced to embed low-rank constraints into the deep learning model layers for fine-grained feature extraction, thus improving structural fidelity. The proposed Bayesian Adaptive Fractional Ridge Regression module, based on singular value space, dynamically adjusts the regularization parameters, significantly enhancing the decoder’s generalization ability under complex stimulus conditions. The constructed Dynamic Confidence Adaptive Diffusion Model module incorporates a confidence network and time decay strategy, dynamically adjusting the semantic injection strength during the generation phase, further enhancing the details and naturalness of the generated images. **Results**: The proposed DCBAN method is applied to the NSD, outperforming state-of-the-art methods by 8.41%, 0.6%, and 4.8% in PixCorr (0.361), Incep (96.0%), and CLIP (97.8%), respectively, achieving the current best performance in both structural and semantic fMRI visual image reconstruction. **Conclusions:** The DCBAN proposed in this thesis offers a novel solution for reconstructing visual images from fMRI signals, significantly enhancing the robustness and generative quality of the reconstructed images.

## 1. Introduction

Reconstructing visual images from fMRI signals [1] refers to the process of using functional magnetic resonance imaging (fMRI) data from the brain to establish complex computational models that can represent the content of visual stimuli, thereby reconstructing the corresponding visual image. Compared to other neuroimaging techniques such as electroencephalography (EEG), positron emission tomography (PET), and near-infrared spectroscopy (NIRS), fMRI data acquisition offers advantages such as non-invasiveness and high spatial resolution. The method of reconstructing visual images from fMRI brain signals can be applied in developing brain–machine interfaces to provide real-time communication and control for paralyzed patients [2], uncovering the encoding mechanisms of human visual cognition to design machine vision systems with enhanced human-like perceptual capabilities [3], understanding the neural activity patterns of mental illnesses for early diagnosis and personalized treatment [4], analyzing brain responses to detect lies and improve the fairness of judicial processes [5], and investigating and identifying the neural activity patterns of the brain during dreams or imagery generation to advance gaming and human–computer interaction [6,7]. It can also be used to monitor shifts in attention trajectories to optimize teaching methods [8], among other fields. However, research in this area still faces numerous challenges that need to be addressed, such as individual differences in neural activity, limitations in the spatial and temporal resolution of fMRI data, and noise interference in the decoding process. Consequently, fMRI brain signal decoding for visual image reconstruction has become a major research focus.

Based on the differences in feature extraction methods and model architectures, fMRI brain signal decoding for visual image reconstruction can be broadly classified into two categories: traditional machine learning-based methods and deep learning-based methods.

In traditional machine learning-based methods, researchers primarily utilize classical machine learning algorithms to establish a linear mapping relationship between fMRI signals and manually designed visual image features for primary visual decoding [9,10,11,12]. These methods are limited to predicting or classifying certain stimuli or features, or identifying specific images seen by the observer; however, they fail to reconstruct the visual images.

In contrast, deep learning-based fMRI brain information decoding for visual image reconstruction benefits from its multi-layered architecture and automatic feature learning and representation capabilities. This enables the capture of complex nonlinear relationships between fMRI data and images, resulting in more accurate visual image reconstruction. These methods can be further classified into non-generative and generative approaches based on their image processing and generation strategies.

Non-generative methods primarily construct cross-modal mapping models between fMRI voxel patterns and predefined visual features. They decode and perform similarity matching or reconstruct rough visual images within a limited set of visual images. However, such methods [13,14,15] lack generalizability and robustness, and they are unable to generate novel, unseen images to decode complex brain activities such as imagination or dreams. This limitation significantly hinders their applications in fields like brain–computer interfaces, personalized healthcare, and virtual reality, where diverse and dynamic demands exist.

In comparison, generative methods aim to generate new visual images from fMRI data by learning the distribution and features of the data. These methods excel in more complex and diverse tasks, such as when patients attempt to convey new intentions or thoughts through a brain–computer interface system, and they can meet the ever-changing demands of the real world. Common generative models include encoder–decoder architectures, Generative Adversarial Networks (GANs), and diffusion models. Models like Loop-Enc-Dec and DA-HLGN-MSFF, which employ encoder–decoder structures to drive fMRI decoding for visual image reconstruction [16,17,18,19,20,21,22], perform reasonably well in visual image reconstruction. However, they underperform in image generation quality, noise handling, and generalization abilities. This is because the simple mapping method of encoder–decoder architectures lacks the ability to model latent features at multiple scales and layers. Generative Adversarial Networks (GANs), with their superior image generation capabilities and excellent performance in handling diverse data distributions, have been progressively introduced to fMRI brain information decoding and visual image reconstruction tasks [23,24,25,26,27,28,29,30,31]. By utilizing adversarial training, GANs optimize the details of fMRI decoded images, particularly improving textures and local structures. Compared to traditional encoder–decoder models, GANs are better suited for managing the fine details of fMRI brain information decoding and visual image reconstruction. However, the adversarial structure of GANs can lead to unstable training processes, and at times, the generator may focus on generating specific patterns, which limits the ability of GANs to efficiently decode fMRI data into high-quality images. In contrast to GAN-based methods, diffusion models can reconstruct high-quality visual images while avoiding mode collapse and offering more stable training. Consequently, numerous diffusion-based generative methods have emerged [32,33,34,35,36,37,38,39,40,41,42]. Latent Diffusion Models (LDM), which rely on latent space diffusion for reconstruction [43], only require a simple linear mapping from fMRI data to latent representations within LDM, enabling high-resolution visual image reconstruction with high semantic fidelity. However, the reconstructed images may lack rich detail. With the advancement of deep learning techniques, generative methods have achieved significant improvements in the visual quality, structural consistency, and semantic fidelity of reconstructed images, with diffusion-based models showing particularly impressive results.

Diffusion models in deep learning generative methods show significant advantages in fMRI brain information decoding and visual image reconstruction, particularly outperforming GAN-based methods in visual image generation quality, pattern diversity, and training stability. The step-by-step denoising process effectively avoids mode collapse, ensuring generated images meet high-resolution and detail consistency requirements. Additionally, these models demonstrate strong adaptability and generalizability for cross-modal alignment. Current methods like MindDiffuser [34], LDM [43], STTM [40], and MindEye2 [44] are now mainstream technologies in this field. However, the diffusion model-driven methods listed still face challenges, such as insufficient stability in feature extraction under noisy environments, poor regularization adaptability during decoding, and rigid semantic condition fusion in the image generation phase. These issues limit the further improvement of decoding accuracy and reconstructed image quality.

To address these challenges, this study proposes a Dynamic Confidence Bayesian Adaptive Network (DCBAN) framework, which targets three areas within the existing research: high structural fidelity, strong generalization, and high naturalness in reconstructed images. The four contributions of this model are:

(1) DCBAN is an end-to-end dynamic confidence Bayesian adaptive diffusion model network for fMRI brain signal decoding and visual image reconstruction. Experiments demonstrate state-of-the-art accuracy and generation quality in both tasks.
(2) Deep Nested Singular Value Decomposition (DeepSVD) is introduced for robust low-rank feature extraction. By embedding the low-rank structure into each layer of the neural network, it suppresses noise and enhances the model’s ability to represent complex structures, improving the structural fidelity of the reconstructed image.
(3) Bayesian Adaptive Fractional Ridge Regression (BAFRR), based on singular value space preprocessing, adaptively adjusts the regularization level. This adaptability accommodates the complexity of different visual stimuli, improving the model’s decoding accuracy and generalization ability.
(4) The Dynamic Confidence Adaptive Diffusion Model (DCAF) predicts the reliability of decoding features dynamically. It considers the dynamic characteristics of different decoding features during visual image reconstruction, significantly enhancing the diversity and naturalness of decoded visual images in complex scenarios.

The remainder of this paper is organized as follows. Section 2 presents the experimental dataset, along with the overall framework and key modules of the proposed DCBAN model. Section 3 describes the experimental environment, experimental design, and results. Section 4 provides a discussion of the experimental analysis. Section 5 concludes the paper and discusses directions for future work.

## 2. Materials and Methods

### 2.1. Dataset

We conducted experiments using visual images and the fMRI neural response features induced by these images in the Natural Scenes Dataset (NSD) [45]. NSD is a large-scale, high-resolution functional imaging project of the human visual cortex, designed to systematically record the neural responses of the human brain to visual image stimuli. This dataset includes 7T fMRI scan data from 8 subjects (subj01–subj08), with each subject completing more than 30 h of image viewing tasks over approximately 30–40 sessions. During the experiment, subjects viewed 10,000 natural images from the Microsoft COCO dataset, of which 9000 images were unique to each subject (unique9000) and 1000 images were shared across all subjects (shared1000). Each image was repeated an average of three times, with each subject being presented with more than 30,000 image instances in total. The entire dataset comprises approximately 213,000 fMRI trial-level measurements. The fMRI data were collected at a voxel resolution of 1.8 mm and a TR of 1.6 s. Neural responses were recorded in the form of blood-oxygen-level-dependent (BOLD) signals, which were then transformed into time-series data. These signals were modeled using a generalized linear model (GLM), and the GLM denoising and ridge regression strategies were applied to obtain the beta estimates corresponding to each stimulus.

To reasonably divide the training, testing, and validation sets, the dataset was partitioned based on the sharedix labeling information provided by the NSD official source. All images in the dataset belonging to the sharedix category (i.e., shared1000) were used as the standardized test set, while the remaining unique images presented to the participants in the current experiment (unique9000) served as the training set.

To strictly prevent any potential data leakage risks, this study implemented complete isolation between the training and test sets at the image level during the data preprocessing phase, ensuring that no sample overlap existed between them.

### 2.2. Model Overview

**Figure 1:**
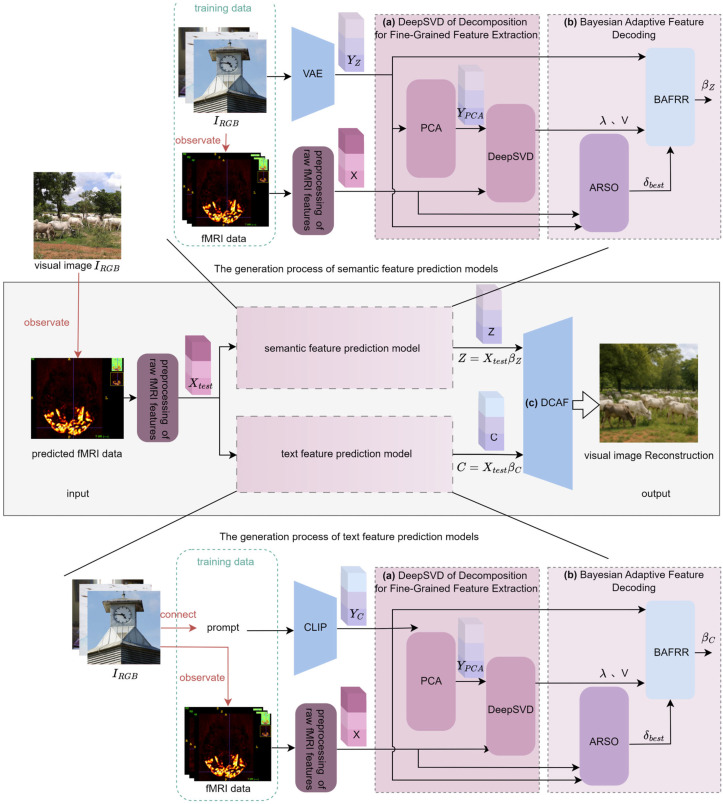
DCBAN model architecture. (**a**) Deep Nested Singular Value Decomposition for Fine-Grained Feature Extraction: The visual image features $Y_{Z}$ or $Y_{C}$ are dimension-reduced using Principal Component Analysis (PCA) to obtain $Y_{PCA}$, and the fMRI data is preprocessed to obtain the fMRI feature X. These two are jointly input into the Deep Nested Singular Value Decomposition (DeepSVD) module, where strongly correlated singular values I and singular value vectors V are extracted (Section 2.3). (**b**) Bayesian Adaptive Feature Decoding: The strongly correlated singular values I drive the construction of a Bayesian Adaptive Fractional Ridge Regression (BAFRR) model. Under the guidance of the fractional score $\delta_{best}$ from the Automated Regularization Score Optimization (ARSO) mechanism, the optimal ridge regression coefficients $\beta_{Z}$ or $\beta_{C}$ are solved, which decodes the predicted fMRI features into visual features *Z* and semantic features *C* (Section 2.4). (**c**) Dynamic Confidence Adaptive Fusion diffusion model (DCAF): The decoded features are then input into the Dynamic Confidence Adaptive Fusion diffusion model, which decodes the predicted fMRI data $X_{test}$ to reconstruct the visual image (Section 2.5).

The proposed DCBAN algorithm framework is shown in Figure 1. Any fMRI data acquired during the observation of a visual image $I_{RGB}$ is used as the test data input for the proposed DCBAN model. After preprocessing the raw fMRI features, the resulting fMRI feature $X_{test}$ is separately input into the semantic feature prediction model and the text feature prediction model to obtain semantic features Z and text features C, respectively. Both features are then input into the Dynamic Confidence Adaptive Fusion diffusion model (DCAF) to reconstruct the visual image corresponding to the fMRI data. As shown in Figure 1, the training structures of the semantic feature prediction model and the text feature prediction model are essentially the same. The primary difference lies in the data preprocessing related to the encoding features extracted from the training data. In the former model, the training data consists of visual images and fMRI data, where the visual images are encoded via a Variational Autoencoder (VAE) [43] to obtain the visual image semantic features $Y_{Z}$. In the latter model, the training data consists of the average text annotations (prompts) related to the visual images and the fMRI data, where the prompts are encoded using CLIP [43] to obtain the visual image text features $Y_{C}$. In both prediction model training structures, the preprocessing of raw fMRI features, Deep Nested Singular Value Decomposition for fine-grained feature extraction, and Bayesian adaptive decoding processing are the same. The preprocessing of raw fMRI features is a standard foundational step that uses Generalized Linear Models (GLMs) to fit, denoise, and average the beta estimates corresponding to each viewing of the same image, thereby forming the feature data X for visual image reconstruction. Clearly, the core components of the proposed DCBAN algorithm model are the Deep Nested Singular Value Decomposition for fine-grained feature extraction, Bayesian adaptive feature decoding, and the Dynamic Confidence Adaptive Fusion diffusion model, as described in three parts below.

### 2.3. Deep Nested Singular Value Decomposition for Fine-Grained Feature Extraction in fMRI Data

In the training structure of semantic and text features, the process primarily involves dimensionality reduction through principal component analysis (PCA), followed by deep nested singular value decomposition (DeepSVD).

It is well known that the dimensions of visual image features ($Y_{Z}$ or $Y_{C}$) and fMRI data features X do not align. Consequently, these features cannot be input into the subsequent deep nested low-rank decomposition model simultaneously for further processing. To address this, visual image features are first reduced to a lower-dimensional representation $Y_{PCA}$ through PCA. This dimensionality reduction allows the features to match those of the fMRI data, facilitating better alignment and making the visual image reconstruction task more efficient.

**Figure 2:**
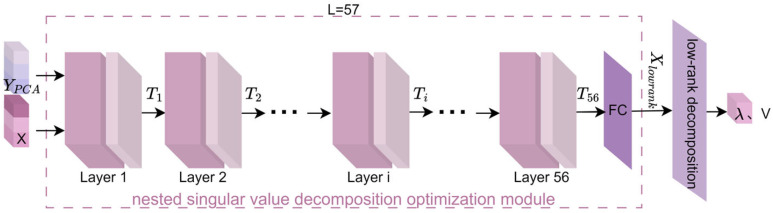
Structure of the DeepSVD Network. $Y_{PCA}$ represents low-dimensional visual features, X denotes fMRI features, and $T_{1}$,$T_{2}$, …, $T_{i}$, …, $T_{56}$ are respectively the outputs of the 1st, 2nd, …, ith, …, 56th layers in nested optimization. FC stands for the fully connected layer. λ refers to the strongly correlated singular values of X, and V is the vector of strongly correlated singular values of X.

Most existing methods use PCA followed by singular value decomposition (SVD). Traditional SVD (a classical low-rank approximation technique) is widely applied in fMRI data processing to remove high-frequency noise and redundant visual features, while extracting fine-grained characteristics. However, SVD is inherently linear, limiting its ability to model nonlinear semantic structures in fMRI data and thus restricting its performance/robustness in image reconstruction. To address this, this study proposes DeepSVD (deep nested singular value decomposition), which improves numerical stability and structural fidelity for more consistent, robust visual image reconstruction under different noise levels. The structure of the proposed DeepSVD network is illustrated in Figure 2 and includes a nested singular value decomposition optimization module and a low-rank decomposition module for extracting singular values and singular vectors.

#### 2.3.1. Nested Singular Value Decomposition Optimization

The nested singular value decomposition optimization module proposed in this study serves as the backbone of the entire network, playing a crucial role in obtaining deeper fMRI feature information within the framework of this paper. The network consists of a total of 57 layers (56 layers of convolutional nested optimization + 1 fully connected layer). Each layer of the neural network incorporates singular value decomposition, which encourages the network to learn representations that align with low-rank structures. As a result, the deep neural network can automatically optimize low-rank representations during the learning process, thereby reducing unnecessary redundant information and improving training efficiency.

**Figure 3:**
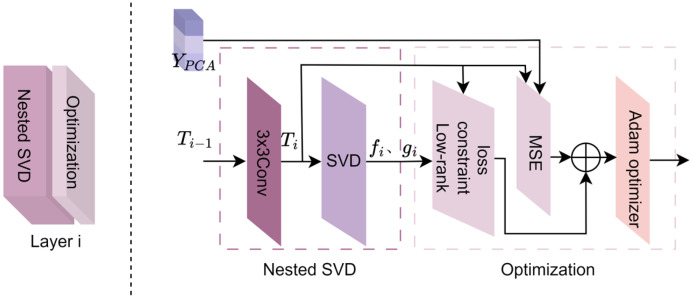
Convolutional nested singular value decomposition of the layer i structure. $T_{i-1}$ is the output of the (i−1)-th layer in nested optimization, $Y_{PCA}$ represents low-dimensional visual features, and 3 × 3 CONV refers to the 3 × 3 convolution layer. SVD stands for singular value decomposition; Loss Constraint Low-rank denotes the calculation of low-rank constraint loss (Step (2)); MSE (Mean Squared Error) refers to the calculation of mean squared error loss; and Adam optimizer refers to the Adam optimizer that updates the parameters of the deep nested singular value decomposition network (Step (6)).

As an example, the operation of the nested singular value decomposition optimization module is illustrated using the layer i (where 1≤i<57) in Figure 3. The process mainly consists of six steps: convolution, SVD, low-rank constraint loss, mean squared error (MSE), loss combination, and Adam optimization.

(1) The output matrix $T_{i-1}$ from the layer i − 1 is convolved with a 3 × 3 filter to obtain $T_{i}$.
(2) Singular value decomposition (SVD) is performed on $T_{i}$, resulting in the left and right singular vectors $f_{i}$ and $g_{i}$, respectively.
(3) To further enhance the independence and effectiveness of feature representation, the singular vectors $f_{i}$ and $g_{i}$ at layer i are subjected to stronger constraints by the low-rank constraint objective function $L_{rank}$. The low-rank constraint objective function $L_{rank}$ consists primarily of the signal matching term, regularization term, and orthogonality constraint term [46], as shown in Equation (1).
  (1) $$L_{rank}=-\beta_{1}\sum_{i=1}^{L}\|g_{i}^{⊤}T_{i}f_{i}{\|}^{2}+\beta_{2}\sum_{i=1}^{L}\|f_{i}{\|}^{2}{\left\|g_{i}\right\|}^{2}+\beta_{3}\sum_{i=1}^{L}\sum_{i^{\prime }=i+1}^{L}\langle f_{i},f_{i^{\prime }}\rangle \langle g_{i},g_{i^{\prime }}\rangle$$

In this context, L represents the number of nested optimization layers. $f_{i}$/$f_{i^{\prime }}$, $g_{i}$/$g_{i^{\prime }}$ refer to the output matrices $T_{i}$/$T_{i+1}$ that undergo Singular Value Decomposition to extract the left and right singular vectors. $\beta_{1}$, $\beta_{2}$, $\beta_{3}$ are weight parameters that balance the contributions of each term. $\left\|\ldots \right\|$ represents the L2 norm (Euclidean norm) of a vector, which is used to measure the length of the vector in Euclidean space.

The first term in Equation (1), $-\beta_{1}\sum_{i=1}^{L}\|g_{i}^{⊤}T_{i}f_{i}{\|}^{2}$, is used to maximize the alignment between $g_{i}$ and $T_{i}$$f_{i}$, thereby preserving the principal information of the input feature X. $g_{i}^{⊤}T_{i}f_{i}$ is the inner product of vectors, as shown in Equation (2).

(2) $$g_{i}^{⊤}T_{i}f_{i}=\|g_{i}\|\cdot \|T_{i}f_{i}\|\cdot \cos\theta$$

Here, θ denotes the angle between $g_{i}$ and $T_{i}$$f_{i}$, so a larger value of $\beta_{1}\sum_{i=1}^{L}\|g_{i}^{⊤}T_{i}f_{i}{\|}^{2}$ indicates better alignment between $g_{i}$ and $T_{i}$$f_{i}$.

The second term in Equation (1), $\beta_{2}\sum_{i=1}^{L}\|f_{i}{\|}^{2}\|g_{i}{\|}^{2}$, imposes a penalty on the magnitudes of $f_{i}$ and $g_{i}$, thereby suppressing their increase in the first item, which merely magnifies the modulus of $g_{i}$ and $f_{i}$. This helps avoid the instability of the model caused by excessive $L_{rank}$.

The third term, $\beta_{3}\sum_{i=1}^{L}\sum_{i^{\prime }=i+1}^{L}\langle f_{i},f_{i^{\prime }}\rangle \langle g_{i},g_{i^{\prime }}\rangle$, enforces the orthogonality of the singular vectors, where $\langle f_{i}/g_{i},f_{i^{\prime }}/g_{i^{\prime }}\rangle$ represents the inner product between $f_{i}$/$g_{i}$ and $f_{i^{\prime }}$/$g_{i^{\prime }}$. The more orthogonal the vectors are, the smaller the inner product, which encourages greater independence among the singular vectors. This reduces redundancy and improves the model’s ability to generalize.

(4) The Mean Squared Error (MSE) layer, as shown in Equation (3), calculates the error between the network output $T_{i}$ and the target matrix $Y_{PCA}$ by computing the squared difference between the two and averaging over all matrix elements.
  (3) $$L_{MSE}=\frac{1}{L}\sum_{i=1}^{L}{\left(T_{i}-Y_{PCA}\right)}^{2}$$
(5) The global loss term $L_{total}$ not only optimizes the overall performance of the network but also applies fine-grained low-rank control at the local level. It is calculated by combining the low-rank constraint objective function $L_{rank}$ with the mean squared error $L_{MSE}$, as shown in Equation (4).

(4) $$L_{total}\;=\;L_{rank}\;+\;\varepsilon L_{MSE}$$

where $L_{rank}$ serves as a structured regularization term that measures the error in the low-rank expansion of the output matrix. The MSE term $L_{MSE}$ acts as a task-driven supervisory term, comparing the error between the output matrix $T_{i}$ and the target matrix $Y_{PCA}$. The inclusion of ε establishes a quantifiable, adjustable, and interpretable trade-off between information retention and redundancy suppression. The combination of these terms ensures that DeepSVD maintains concise feature representations while preserving critical information, thereby enhancing the numerical stability of training and the interpretability of the model.

When the global loss reaches the expected threshold, it indicates that the network has been trained effectively and that the model is capable of efficiently extracting key features from the fMRI data and accurately reconstructing visual images. If the global loss does not meet the predetermined standard, it suggests that the model may be experiencing overfitting, underfitting, or inadequate feature extraction. In such cases, it may be necessary to adjust parameters such as the learning rate, optimization strategy, or regularization parameters, or to retrain the model to further optimize the loss function. By dynamically adjusting these parameters, the model’s performance can be progressively improved until the global loss reaches an optimal level.

(6) The Adam optimizer [47] updates the parameters of the deep nested Singular Value Decomposition (DeepSVD) network, as shown in Equation (5).
  (5) $$\theta_{i}\;=\;\theta_{i-1}-\eta \cdot \frac{{\hat{m}}_{i}}{\sqrt{{\hat{v}}_{i}}\;+\;\epsilon }$$

Here, $\theta_{i-1}$ represents the parameters of the layer *i* − 1 of the network, and η denotes the learning rate. ${\hat{m}}_{i}$ and ${\hat{v}}_{i}$ represent the momentum estimate and the variance estimate of the gradient, respectively. ϵ is a very small constant used to prevent division by zero errors.

Within the nested optimization framework, the model calculates the local gradients for each network layer through the backpropagation mechanism. The local gradients obtained are then utilized by the Adam optimizer to update the weights of each layer, enabling global joint optimization. This strategy not only ensures efficient training of the network under the low-rank constraints at each layer but also effectively drives the minimization of the overall loss function. Through the iterative optimization of all layer parameters, the network ultimately produces an optimal solution matrix $X_{\mathrm{lowrank}}$ that is structurally sparse and compact in representation.

#### 2.3.2. Calculation of Singular Values and Vectors

$Y_{PCA}$ and X undergo L-layer nested optimization to output the optimal matrix $X_{\mathrm{lowrank}}$. This matrix is then subjected to low-rank decomposition, yielding the final strongly correlated singular values λ and the singular vectors V.

(1) Computation of Strongly Correlated Singular Values: The matrix λ is computed by calculating the norm of each column of the matrix $X_{\mathrm{lowrank}}$, as shown in Equation (6).

(6) $$\lambda \;=\;{\left\|X_{lowrank}^{\left(i\right)}\right\|}^{2}$$

where $X_{lowrank}^{\left(i\right)}$ represents the i-th column of the matrix $X_{\mathrm{lowrank}}$, and ${\left\|\cdot \right\|}^{2}$ denotes the Euclidean norm.

(2) Computation of Singular Vectors: The matrix V is derived by normalizing the output matrix $X_{\mathrm{lowrank}}$, as shown in Equation (7).

(7) $$V\;=\;\frac{X_{lowrank}}{{\left\|X_{lowrank}\right\|}_{2}}$$

### 2.4. Bayesian Adaptive Feature Decoding

**Figure 4:**
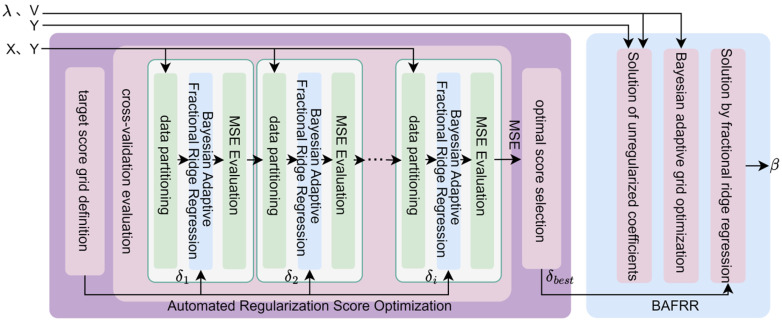
Bayesian adaptive decoding process. X refers to fMRI features, and Y denotes visual image features. λ refers to the strongly correlated singular values of X, and V is the vector of strongly correlated singular values of X. $\delta_{1}$, $\delta_{2}$, …, $\delta_{i}$ are respectively the 1st, 2nd, …, ith target scores in the target score grid, and $\delta_{\mathrm{best}}$ is the optimal target score. β represents the coefficient solution of BAFRR.

To address the issue of insufficient generalization capability in traditional ridge regression under high-dimensional fMRI data, this study proposes a Bayesian adaptive feature decoding method, as shown in Figure 4. This method combines an automated regularization score optimization approach (Section 2.4.1) with the training data X and Y, automatically selecting a score δ($\delta_{1}$,$\delta_{2}$,…,$\delta_{i}$) from a predefined grid and training the Bayesian Adaptive Fractional Ridge Regression (BAFRR) (Section 2.4.2) for evaluation to obtain the optimal score $\delta_{\mathrm{best}}$. This value, along with the strongly correlated singular values and singular vectors, is then used to train the Bayesian Adaptive Fractional Ridge Regression to obtain the solution β, which is used to predict high-precision visual image features (Section 2.4.3).

#### 2.4.1. Automated Regularization Score Optimization

Ridge regression is widely used to establish the mapping between fMRI data and visual stimulus features, as it effectively mitigates the overfitting problem that arises due to the high dimensionality of fMRI data and low sample sizes. However, the global regularization approach in ridge regression cannot assign differentiated regularization weights to features with different dimensions when handling complex stimulus features, leading to issues such as information loss or underfitting of specific features. The Fractional Ridge Regression (FRR) method combines the stability of ridge regression with a more granular regularization strategy. By introducing non-uniform regularization weights for specific features, it enables the model to capture complex stimulus features while maintaining robustness to noise, thereby improving decoding performance and model generalization. However, in the existing fractional ridge regression method [46], the choice of the fractional parameter is typically reliant on manual adjustment, a process that is both cumbersome and uncertain. To address this issue, the Automated Regularization Score Optimization for Ridge Regression (ARSO) method is proposed. This method efficiently selects the optimal ridge regression regularization score by automatically searching for the appropriate regularization level based on an optimized target score grid. The method consists of three main steps: target score grid definition, cross-validation evaluation, and optimal score selection, as shown on the left side of Figure 4.

(1) target score grid definition

To facilitate effective adjustment of the regularization level, a target score grid Λ containing various regularization levels is defined, as shown in Equation (8). The grid starts at $\delta_{\min}=0$ (representing no regularization in the ridge regression model) and ends at $\delta_{\max}=1$ (representing extremely strong regularization in the ridge regression model), with a step size of Δδ=0.05.

(8) $$\Lambda =\{\delta_{i}|\delta_{i}=\delta_{\min}+i\cdot \Delta \delta ,i=0,1,2\ldots ,N\}$$

Here, $\delta_{i}$ denotes the *i*-th score in the target score grid, where each score represents the ratio between the norm of the target coefficient and the norm of the least squares (OLS) solution. The target score grid spans the entire range from weak to strong regularization. A smaller step size increases the likelihood that the model will achieve precise regularization, though it incurs a higher computational expense.

(2) cross-validation evaluation

fMRI data features X and visual image features Y (either visual image features $Y_{Z}$ or visual image text features $Y_{C}$) are used as the training dataset. For each score value $\delta_{i}$ in the grid Λ, a *k*-fold cross-validation is applied to evaluate the model on the training data. The cross-validation procedure primarily involves three steps: data partitioning, training the Bayesian Adaptive Fractional Ridge Regression model (detailed in Section 2.4.2), and MSE evaluation.

In each cross-validation fold, the training dataset is divided into *k* mutually exclusive subsets, with *k* − 1 subsets used for training the Bayesian adaptive grid-optimized ridge regression model, and the remaining subset used for validating the model’s performance. A model is retrained in each fold and evaluated on the corresponding validation set.

Mean Squared Error (MSE) is used to evaluate the average squared error between the predicted and true values during each fold. A smaller MSE indicates better model performance, as shown in Equation (9).

(9) $$MSE_{i}=\frac{1}{\left|D_{i}\right|}{\sum_{j\in D_{i}}\left(Y_{i}-X_{j}^{T}{\hat{\beta }}_{\delta_{i}}\right)}^{2}$$

where $MSE_{i}$ represents the average prediction error of the model in the *i*-th fold of the cross-validation. $D_{i}$ is the validation set for the *i*-th fold of the cross-validation. $\left|D_{i}\right|$ is the number of samples in the *i*-th validation set. $Y_{i}$ is the target variable visual image feature ($Y_{Z}$ or $Y_{C}$) in the *i*-th fold of the cross-validation. $X_{j}$ is the *j*-th fMRI data feature vector in the validation set $D_{i}$. ${\hat{\beta }}_{\delta_{i}}$ is the estimated ridge regression coefficient for the *i*-th score value $\delta_{i}$ in the target score grid based on the Bayesian adaptive grid-optimized ridge regression model, and $X_{j}^{T}{\hat{\beta }}_{\delta_{i}}$ is the predicted visual image feature $Y_{i}$ ($Y_{Z}$ or $Y_{C}$) for the *j*-th fMRI data feature vector by the current ridge regression model.

The process is repeated *k* times to ensure that each subset serves as the validation set once. The *k* validation errors are then averaged to serve as the evaluation metric for that particular score value. This approach allows for the assessment of the model’s generalization ability for each score value and helps prevent evaluation bias that could arise from random data partitioning.

(3) optimal score selection

After completing all the cross-validation steps and obtaining *k* evaluation MSE values, the ARSO method proposed in this study selects the score δ corresponding to the smallest MSE as the optimal target score $\delta_{\mathrm{best}}$. This optimal score represents the best regularization level for the ridge regression model, effectively balancing overfitting and underfitting. As the final regularization target, this score is used to retrain the Bayesian adaptive grid-optimized ridge regression model using the complete training set. The final ridge regression solution is then computed and utilized for subsequent visual image decoding tasks.

#### 2.4.2. Bayesian Adaptive Fractional Ridge Regression

**Figure 5:**
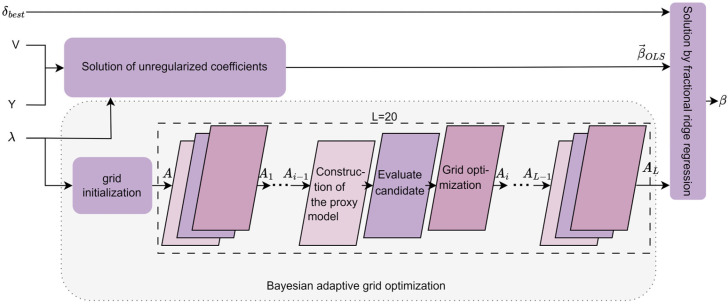
The solution process of Bayesian adaptive grid optimization for ridge regression. Y refers visual image features. λ refers to the strongly correlated singular values of X, and V is the vector of strongly correlated singular values of X. $\delta_{\mathrm{best}}$ is the optimal target score. A is the search grid, and $A_{1}$, …, $A_{i-1}$, $A_{i}$, …, $A_{L}$ are respectively the 1st, …, (i−1)th, ith, …, Lth layer output grids of Bayesian adaptive iteration. ${\tilde{\beta }}_{OLS}$ is the unregularized coefficient solution. β represents the coefficient solution of BAFRR.

The traditional fixed *α*-grid method is commonly used in ridge regression for the search of regularization parameters. However, due to its inability to dynamically adapt to the distribution of data features, it often results in grids that are either too sparse or too dense, leading to wasted computational resources or the omission of the optimal parameter choice. To address this issue, the Bayesian Adaptive Fractional Ridge Regression (BAFRR) method has been proposed. The specific process is illustrated in Figure 5 and is divided into three main steps: solving for the unregularized coefficients, singular value-driven Bayesian adaptive grid optimization, and solving for the optimal target score using fractional ridge regression. As the core of the method, Bayesian optimization dynamically adjusts the grid precision using a Gaussian process surrogate model and acquisition function, iterating *L* times (where *L* = 20) to optimize *α*, thereby enhancing both search efficiency and generalization ability.

(1) Solution of unregularized coefficients

This process utilizes Singular Value Decomposition (SVD) to map the high-dimensional problem into a low-dimensional singular value space and solves for the unregularized coefficient solution, which serves as a reference for subsequent regularization.

To map the high-dimensional problem into the low-dimensional singular value space, the transpose of the singular vector matrix V, obtained from the DeepSVD, is multiplied by the visual image feature Y (either $Y_{Z}$ or $Y_{C}$), generating the transformed target variable $\tilde{Y}$, as shown in Equation (10).

(10) $$\tilde{Y}\;=\;V^{T}Y$$

This $\tilde{Y}$, along with the singular values λ obtained from the DeepSVD, solves the ordinary least squares (OLS) problem through simple scalar multiplication, resulting in the unregularized coefficient solution ${\tilde{\beta }}_{OLS}$ as shown in Equation (11).

(11) $${\tilde{\beta }}_{OLS}\;=\;S^{-2}(\lambda ⊙\tilde{Y})$$

Here, ⊙ denotes element-wise multiplication. S represents the diagonal vector of singular values (i.e., the set of singular values), and $S^{-2}$ refers to the inverse of the square of each singular value, which is used to scale the solution in the singular value space. ${\tilde{\beta }}_{OLS}$ corresponds to the form of the original OLS solution in the singular value space, representing the OLS coefficients after dimensionality reduction and rotation by the singular value matrix. ${\tilde{\beta }}_{OLS}$ forms the basis for the linear mapping between the strongly correlated singular values λ of the fMRI data features X and the visual image features Y ($Y_{Z}$ or $Y_{C}$), and provides a controllable reference point for subsequent regularization adjustments, helping fractional ridge regression optimize the model’s complexity and generalization ability.

(2) Bayesian adaptive grid optimization

Bayesian optimization is a black-box optimization method used to optimize complex, expensive, or noisy functions, and it is widely applied in hyperparameter tuning and experimental design. The process involves constructing a surrogate model and using uncertainty to guide the search, gradually converging to the global optimum. This study introduces Bayesian adaptive grid optimization, which primarily consists of two steps: grid initialization and adaptive iteration.

① Grid initialization

The initial candidate regularization parameter is generated through a logarithmic spacing sampling method to form the search grid, which is independent of the previous score grid, as shown in Equation (12).

(12) $$A=(\alpha_{k}|\alpha_{k}=10^{\log_{10}(SMALL\text{\_}BIAS\cdot \sigma_{\min}^{2})+k\cdot \Delta A},\;k=0,1,\ldots ,N)$$

Here, $N=\frac{\log_{10}(BIG\text{\_}BIAS\cdot \sigma_{\max}^{2})-\log_{10}(SMALL\text{\_}BIAS\cdot \sigma_{\min}^{2})}{\Delta }$, $\sigma_{\max}$ and $\sigma_{\min}$ are the maximum and minimum singular values obtained by DeepSVD. SMALL_BIAS and BIG_BIAS are expansion factors. ΔA represents the sampling step size in logarithmic space. This logarithmic spacing sampling method is particularly suitable for optimization problems involving regularization parameters, especially when the parameter values vary significantly across orders of magnitude.

During the Bayesian Adaptive Grid Optimization (BAGO) process, the formulation of the objective function is central to the optimization framework. To optimize the sparse and dense distribution of the regularization parameter α within the search grid A (Equation 12), this study utilizes the prediction error of the model on the validation set as the optimization goal. Specifically, the mean squared error (MSE) is employed as the metric, as shown in Equation (13).

(13) $$MSE(\alpha )=\frac{1}{n}\sum_{i=1}^{n}{\left(y_{i}-{\hat{y}}_{i}(\alpha )\right)}^{2}$$

where $y_{i}$ is the true target value of the i-th sample in the visual image feature set Y ($Y_{Z}$ or $Y_{C}$), and ${\hat{y}}_{i}(\alpha )$ is the prediction made by the ridge regression model, which is trained using the current candidate? α from the search grid A on the validation set. n is the number of samples.

② adaptive iteration

The Bayesian Adaptive Grid Optimization (BAGO) method we propose approximates the objective function of Bayesian optimization based on a surrogate model. It evaluates candidate values of α with higher uncertainty by selecting them from the search grid A (Equation 12) through the acquisition function. The search grid A is iteratively optimized for *L* iterations (*L* = *N*), refining its sparse and dense distributions. This approach adaptively refines grid B in areas with higher feature distribution density to capture precise solutions while increasing the step size in sparse regions to reduce unnecessary computations. This significantly improves search efficiency and generalization ability. The Bayesian adaptive grid optimization module undergoes *L* iterations (*L* = *N*), and the working principle of the *i*-th iteration is explained with the following main steps: constructing the proxy model, evaluating candidate α, and optimizing the grid, as shown in Figure 5.

(a) Construction of the proxy model

In each iteration, the surrogate model is constructed based on the previously optimized search grid $A_{i-1}$. The Gaussian Process (GP) is employed as the surrogate model in Bayesian optimization to approximate the objective function. Let $A_{i-1}$ consist of n regularization parameters $\left\{\alpha_{1},\alpha_{2},\ldots ,\alpha_{i},\ldots ,\alpha_{n}\right\}$ and their corresponding validation error observations $\left\{y_{1},y_{2},\ldots ,y_{i},\ldots ,y_{n}\right\}$ (i.e., the average error observed on the validation set after training the fractional ridge regression with the corresponding α) as the training set. The Gaussian process model provides predictions in terms of the mean $m\left(\alpha_{i}\right)$ and the standard deviation $\sigma (\alpha_{i})$ for each new parameter point $\alpha_{i}$ to be evaluated, where $m\left(\alpha_{i}\right)$ represents the expected performance and $\sigma (\alpha_{i})$ quantifies the uncertainty for that point.

Here, the prediction mean [48] $m\left(\alpha_{i}\right)$ is given by Equation (14).

(14) $$m(\alpha_{i})\;=\;r{\left(\alpha_{i}\right)}^{T}{\left[R\;+\;\sigma_{n}^{2}I\right]}^{-1}y_{i}$$

The prediction standard deviation [48] $\sigma (\alpha_{i})$ is given by Equation (15).

(15) $$\sigma^{2}(\alpha_{i})\;=\;k(\alpha_{i},\alpha^{\prime })\;-\;r{\left(\alpha_{i}\right)}^{T}{\left[R\;+\;\sigma_{n}^{2}I\right]}^{-1}r(\alpha_{i})$$

Here, $k(a_{i},a^{\prime })$ denotes the autocovariance term, which reflects the fluctuations of function values under prior assumptions. $r(\alpha_{i})$ is the covariance vector between the point being evaluated and the already assessed parameters in the training set; *R* is the covariance matrix formed by the known parameter points; $\sigma_{n}^{2}$ represents the observation error term, modeling the noise uncertainty in the objective function, and I is the identity matrix. This model enables continuous and differentiable estimation of the performance of any candidate parameter point within the current grid by jointly modeling the existing data.

The covariance function between the new regularization parameter $\alpha_{i}$ and the already evaluated parameters $\alpha^{\prime }$ is selected using the squared exponential kernel (Squared Exponential Kernel) [49], as given by Equation (16).

(16) $$k(\alpha_{i},\alpha^{\prime })\;=\;\upsilon^{2}\exp\left(-\frac{{\left(\alpha_{i}\;-\;\alpha^{\prime }\right)}^{2}}{2l^{2}}\right)$$

Here, $\upsilon^{2}$ controls the fluctuation of the function values, while the length scale l determines the rate of change in the function values within the input space.

(b) Evaluate candidate α

After the surrogate model is determined, this study uses the Expected Improvement (EI) function as the acquisition function for Bayesian optimization. The magnitude of the EI value measures the potential performance improvement of a parameter point $\alpha_{i}$ relative to the smallest validation error observed among all current parameter points. In other words, the larger the EI value, the more likely the parameter point will achieve the smallest validation error compared to the already observed points, thus indicating greater potential for performance improvement. The EI value [50] for a candidate parameter $\alpha_{i}$ in the set $A_{i-1}$= $\left\{\alpha_{1},\alpha_{2},\ldots ,\alpha_{i},\ldots ,\alpha_{n}\right\}$ is calculated, with the definition provided in Equation (17).

(17) $$EI(\alpha_{i})\;=\;(m(\alpha_{best})\;-\;m(\alpha_{i}))\cdot \Phi (Z)\;+\;\sigma (\alpha_{i})\cdot \phi (Z)$$

where

$$Z\;=\;\frac{m(\alpha_{best})\;-\;m(\alpha_{i})}{\sigma (\alpha_{i})}$$

$m(\alpha_{best})$ represents the smallest validation error among all currently observed parameter points. $\alpha_{best}$ is the parameter in $A_{i-1}$ that minimizes the predicted mean (as shown in Equation (14)), and $m(\alpha_{i})$ and $\sigma (\alpha_{i})$ are the predicted mean and standard deviation of the surrogate model for the current $\alpha_{i}$, respectively. Φ(·) and ϕ(·) represent the cumulative distribution function and probability density function of the standard normal distribution, respectively.

The EI values for all parameters α in set $A_{i-1}$ are calculated. The point $\alpha_{cur}$ with the highest EI value is selected as the next evaluation point. If its predicted mean $m(\alpha_{cur})$ is smaller than $m(\alpha_{best})$, then $\alpha_{best}$ is updated to $\alpha_{cur}$.

(c) Grid optimization

During the evaluation of the acquisition function, Bayesian optimization continuously refines the precision of the grid. After the *i*-th Bayesian iteration optimization, grid $A_{i}$ will be adaptively redistributed and adjusted based on $\alpha_{best}$, which is associated with the current minimum predicted mean, as shown in Equation (18).

(18) $$A_{i}\;=\;\left\{\alpha_{k}\left|\alpha_{k}\;=\;\alpha_{best}\;+\;s\cdot \tanh\left(d\cdot \frac{2k\;-\;N}{N}\right),\;\;k\;=\;0,1,\ldots ,N\right.\right\}$$

Here, s is the maximum radius by which the entire grid expands around $\alpha_{best}$. d is the focusing parameter, where a larger value results in points becoming more concentrated around $\alpha_{best}$. tanh(·) is the hyperbolic tangent function, used to achieve non-uniform concentration of grid points around $\alpha_{best}$, with its definition provided in Equation (19).

(19) $$\tanh(x)\;=\;\frac{e^{x}\;-\;e^{-x}}{e^{x}\;+\;e^{-x}}$$

The Bayesian adaptive grid optimization module undergoes *L* = 20 iterations of the above process, resulting in the final optimized grid $A_{L}$.

(3) Solution by fractional ridge regression

According to the various tasks in Bayesian adaptive fractional ridge regression, different scores are selected as the target score δ: during the cross-validation model training task for automated regularization score optimization, score $\delta_{i}$ from the target score grid Λ is chosen as the target score δ; during the calculation of the final BAFRR solution, the optimal score $\delta_{\mathrm{best}}$ selected by ARSO is used as the target score δ. To obtain the regularization parameter $\alpha_{\delta }$ corresponding to the target score δ, an interval is identified in the list $Ρ\{(\alpha_{i},\gamma_{i})\}$ such that the condition $\gamma_{j}\leq \delta \leq \gamma_{j+1}$ is satisfied. The corresponding value of $\gamma_{j}/\gamma_{j+1}$ is associated with $\alpha_{j}/\alpha_{j+1}$ in $Ρ\{(\alpha_{i},\gamma_{i})\}$. The calculation of the regularization parameter $\alpha_{\delta }$ corresponding to the target score δ [51] is presented in Equation (20).

(20) $$\alpha_{\delta }\;=\;\alpha_{j}+(\delta \;-\;\gamma_{j})\cdot \frac{\alpha_{j+1}\;-\;\alpha_{j}}{\gamma_{j+1}\;-\;\gamma_{j}}$$

The scaling factor vector $\frac{\lambda^{2}}{\lambda^{2}\;+\;\alpha_{\delta }}$, obtained by calculating the singular values λ and the regularization parameter $\alpha_{\delta }$ from the deep nested singular value decomposition (DeepSVD) of fMRI data fine-grained feature extraction is used to adjust the coefficients along the principal component directions. This process enhances the model’s stability under high-dimensional data and mitigates the risk of overfitting [43]. This scaling vector is applied to the unregularized least squares (OLS) solution ${\tilde{\beta }}_{OLS}$, resulting in the regularized regression coefficient estimate ${\tilde{\beta }}_{RR}$ [51] in the low-dimensional space, as shown in Equation (21).

(21) $${\tilde{\beta }}_{RR}\;=\;\frac{\lambda^{2}}{\lambda^{2}+\alpha_{\delta }}⊙{\tilde{\beta }}_{OLS}$$

The resulting ridge regression solution ${\tilde{\beta }}_{RR}$ is multiplied by the singular value vector matrix *V*, which is obtained from DeepSVD (calculated in Equation (5)), and transformed back to the original space. This yields the final coefficient solution β [46] for the Bayesian adaptive fractional ridge regression model, as shown in Equation (22).

(22) $$\beta \;=\;V{\tilde{\beta }}_{RR}$$

#### 2.4.3. Semantic Feature Prediction and Text Feature Prediction

In this study, visual images’ semantic features $Y_{Z}$ and text features $Y_{C}$ are used as training labels to train two independent Bayesian adaptive fractional ridge regression models. During the model training phase, the corresponding coefficient solutions $\beta_{Z}$ and $\beta_{C}$ are optimized based on the mapping relationship between the fMRI data features and the training labels. These coefficient solutions are then used to predict the semantic features and text features, respectively. In the model testing phase, the fMRI feature data $X_{\mathrm{test}}$ to be predicted is used with the coefficient solution $\beta_{Z}$ to predict the corresponding semantic feature *Z* for the early visual cortex, as shown in Equation (23).

(23) $$Z\;=\;X_{\mathrm{test}}\beta_{Z}$$

Similarly, the fMRI feature data $X_{\mathrm{test}}$ to be predicted is used with the coefficient solution $\beta_{C}$ to predict the corresponding text feature *C* for the ventral temporal cortex, as shown in Equation (24).

(24) $$C\;=\;X_{\mathrm{test}}\beta_{C}$$

This bimodal decoding method effectively combines image semantic information and latent text features, further revealing the multimodal interaction mechanisms in the brain when processing visual and linguistic information [43].

### 2.5. Dynamic Confidence Adaptive Fusion Diffusion Visual Image Reconstruction

Traditional brain feature decoding methods [41,43] often employ static feature fusion of brain signals and generated priors, injecting the decoded semantic features into the diffusion model through a cross-attention mechanism with fixed weights. These methods lack flexible constraints in the decoding process, leading to semantic distortion or content blurring in the reconstructed images, as well as neural signal noise and semantic representation bias. Additionally, they struggle to adapt to semantic conditions with varying confidence levels and fail to dynamically balance detail fidelity and generative freedom during iterative sampling. Generative freedom, however, allows the model to introduce greater variations in detail and style when generating images or other content, thereby enhancing the complexity and realism of the generated images.

**Figure 6:**
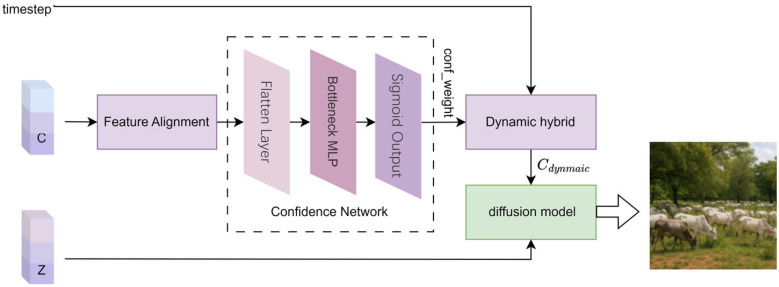
Dynamic confidence adaptive fusion diffusion visual image reconstruction.

To address this issue, this study proposes the Dynamic Confidence Adaptive Fusion (DCAF) model, embedding a confidence network into the fMRI brain signal decoding and visual image generation task. The DCAF model maps high-dimensional text features *C* into dynamic confidence weights through a cascaded dimensionality reduction MLP architecture, thereby suppressing misleading condition injections from low-reliability text features and enhancing noise robustness. Building on this, the DCAF model introduces a time-step-based decay factor to implement progressive dynamic mixing and conditional relaxation. This approach strengthens semantic constraints in the early stages of visual image generation to stabilize content structure while later releasing the model’s priors to enrich the diversity of visual image details. The primary implementation process of the model consists of four steps: feature alignment, confidence-aware condition modulation, dynamic mixing, and diffusion reconstruction. The specific process is illustrated in Figure 6.

(1) Feature Alignment

The text feature *C* is predicted using Bayesian Adaptive Fractional Ridge Regression and serves as a conditional input. To fit the diffusion model’s conditional embedding space, the original text feature *C* is reshaped to its standard output dimension of (77, 768). This dimensionality adjustment retains the structural priors of the text-conditioned diffusion model while ensuring compatibility with the conditional embedding layer of the pretrained diffusion model.

(2) Confidence Network for Dynamic Quantization of Semantic Features

To dynamically quantify the reliability of the semantic features *C*, a lightweight Confidence Network (LCN) is designed. This network primarily consists of a flatten layer, a bottleneck MLP layer, and a sigmoid output layer.

① Flatten Layer: To better adapt the text for processing by the fully connected layer, the feature *C* is flattened from its original shape of (77, 768) into a one-dimensional vector, as shown in Equation (25).

(25) $${Ζ}_{1}\;=\;Flatten\left(C\right)$$

② Bottleneck MLP Layer: This layer primarily consists of a Multi-Layer Perceptron (MLP) and a ReLU activation function for processing one-dimensional vectors, as shown in Equation (26). The one-dimensional text features are compressed into a 128-dimensional bottleneck structure through a fully connected layer. This compression process filters out non-essential noise and reduces the computational load, thereby streamlining the network structure. The ReLU activation function is employed during this process to retain only the positive signals while suppressing the propagation of negative noise, thus adapting to the sparse semantic characteristics of the brain decoding features.

(26) Ζ2 = Relu(MLP(Ζ1))

③ Sigmoid Output Layer: The Sigmoid function processes the data to output a confidence weight conf_weight in the range [0, 1], as shown in Equation (27). This equation quantifies the reliability of the semantic condition. The larger the value of conf_weight, the higher the reliability of the semantic condition. According to Equation (31), different text features from various test data possess different cross-sample dynamic confidence perception weights conf_weight.

(27) $$conf\text{\_}weight\;=\;\sigma ({Ζ}_{2})$$

The network employs a bottleneck MLP (Multilayer Perceptron) layer to filter out redundant noise from the brain decoding features while retaining key semantic information. The ReLU activation function, with its non-exponential operations, requires very few parameters and exhibits low latency, making it suitable for real-time sensitive applications such as brain-machine interfaces. Although the Sigmoid layer involves exponential calculations, it applies only to the final one-dimensional output, rendering its impact on overall latency negligible.

(3) Dynamic Adaptive Control Generation of Text Features

The generation prior refers to the intrinsic generative preferences of the diffusion model learned during the pre-training phase, including image structure, semantic configuration, and modal distribution. This represents the model’s default generative tendency in the absence of semantic guidance (such as text or brain features). In the process of reconstructing visual images using the diffusion model, dynamic confidence perception weights conf_weight (as shown in Equation (27)) are adaptively introduced via the time decay factor and a lightweight confidence network (LCN). This mechanism constructs a flexible fusion path between brain features and the generation prior. As the injection weight of semantic features decreases, the model increasingly relies on its generation prior to completing the image reconstruction, thus maintaining naturalness and structural stability.

The dynamic coupling of confidence perception weights and the time decay factor generates dynamic text features $C_{dynamic}$, which adaptively reduces the risk of misguidance from low-quality inputs. The process of generating these dynamic text features can be represented by Equation (28).

(28) $$C_{dynamic}^{\left(n\right)}\;=\;\xi^{\left(n\right)}\cdot C\;+\;(1\;-\;\xi^{\left(n\right)})\cdot u_{C}$$

Here, *C* represents the text features obtained from the text feature prediction model. $u_{C}$ is the unconditional vector. The dynamic mixed weight $\xi^{\left(n\right)}$ is determined jointly by the confidence weight conf_weight from Equation (31) and the time decay factor time_decay, as shown in Equation (29):

(29) $$\xi^{\left(n\right)}\;\;=\;\;conf\text{\_}weight\;\;\times \;\;time\text{\_}decay(n)$$

In this equation, time_decay is the time decay factor. The time decay factor is a dynamic weight adjustment mechanism based on the timestep iteration number *n*, used to control the gradual relaxation of semantic conditions during the generation process. The core goal is to balance content fidelity in the early stages of visual image generation with the diversity of details in the later stages through an annealing-style constraint strategy.

Let the current timestep iteration be n∈{0,1,…,niter−1}, where niter represent the total number of iterations. The time decay factor is defined as shown in Equation (30).

(30) $$time\text{\_}decay(n)\;=\;max(0,1\;-\;\frac{n}{niter})$$

where the $\frac{n}{niter}$ part increases linearly with the number of iteration steps *n*. As the number of sampling steps increases, the decay factor gradually decreases from 1 to 0. The weight of semantic conditions is smoothly reduced, gradually allowing for greater freedom in generating details.

The time decay factor, through a linear decay strategy, prevents mode collapse due to noisy conditions in the early stages of generation. In the later stages of iteration, the introduction of randomness prevents excessively rigid generation outcomes.

(4) Diffusion Model for Visual Image Reconstruction

The dynamic vector $C_{dynamic}$, obtained from the dynamic confidence adaptive diffusion model (DCAF), is combined with the semantic feature *Z*, predicted by Bayesian adaptive fractional ridge regression. Both are used as inputs to the diffusion model. The diffusion model employs the DDIM accelerated sampling algorithm [4], performing iterative denoising in the latent space to reconstruct the visual image.

## 3. Results

This section may be divided by subheadings. It should provide a concise and precise description of the experimental results, their interpretation, as well as the experimental conclusions that can be drawn.

### 3.1. Experimental Setup

The hardware used in this experiment includes an NVIDIA GeForce RTX 3060 GPU, 64 GB of Random Access Memory (RAM), and a 3.7 TB hard disk drive. The operating environment is Ubuntu 20.04 LTS, with GPU acceleration implemented using CUDA 11.8. Python 3.10 was used as the programming language to implement all experimental code, and the main toolboxes and frameworks utilized include PyTorch 2.0, scikit-learn, NumPy, SciPy, pandas, and pytorch-lightning, among others.

The DCBAN method is primarily divided into three modules: DeepSVD, BAFRR, and DCAF. The hyperparameters of each module, along with the types, quantities, and selection rationale for each hyperparameter, are summarized in Table 1.

### 3.2. Evaluation Metrics

To comprehensively assess the quality of reconstructed images, a set of integrated metrics covering low-level visual similarity and high-level semantic fidelity is employed. These metrics are commonly used in recent fMRI-based image reconstruction studies and offer strong generalizability and comparability.

(1) Low-level visual similarity

Two pixel-level metrics, the pixel-wise Pearson correlation coefficient and the Structural Similarity Index (SSIM), are used to measure the visual consistency between the reconstructed image and the ground truth image.

① Pixel-wise Pearson Correlation (PixCorr) [52]: This metric calculates the linear correlation between the pixel values of two images to reflect the overall visual structural consistency, as shown in Equation (31).

(31) $$PixCorr\;=\;\frac{\sum_{i=1}^{n}\left(x_{i}\;-\;\bar{x}\right)(y_{i}\;-\;\bar{y})}{\sqrt{\sum_{i=1}^{n}{\left(x_{i}\;-\;\bar{x}\right)}^{2}}\cdot \sqrt{\sum_{i=1}^{n}{\left(y_{i}\;-\;\bar{y}\right)}^{2}}}$$

where $x_{i}$ is the *i*-th pixel value of the original image, $y_{i}$ is the *i*-th pixel value of the reconstructed image, $\bar{x}$ and $\bar{y}$ are the mean pixel values of the original and reconstructed images, respectively, and *n* is the total number of pixels in both images.

② Structural Similarity Index (SSIM) [52]: This index evaluates the similarity between the low-level structural features of the reconstructed and ground truth images by considering luminance, contrast, and structural information, as shown in Equation (32).

(32) SSIM(x,y)=l(x,y)·c(x,y)·s(x,y)

Among them, l(x,y), c(x,y), and s(x,y) are, respectively, the luminance comparison, contrast comparison and structure comparison at the pixel point (x,y).

(2) High-Level Semantic Fidelity Evaluation

To comprehensively assess the fidelity of the reconstructed image at different semantic levels, we introduce five types of high-level semantic representations based on visual perception models: AlexNet-based feature similarity (Alex-2/Alex-5), Inception feature similarity (Incep), CLIP semantic embedding similarity (CLIP), and EfficientNet classification accuracy difference (Eff). These visual perception models have been widely used in fMRI brain decoding for evaluating the quality of visual image reconstruction [40,50].

Alex-2/Alex-5 uses a pre-trained AlexNet, extracting features from conv2 (shallow edge perception) and conv5 (mid-level texture perception) to measure the low-level visual structure consistency of reconstructed images. Incep employs 2048-dimensional features from the avgpool layer of Inception-v3 to assess overall image semantic consistency and calculate Fréchet Inception Distance (FID). CLIP uses the image encoder of ViT-L/14 to extract cross-modal semantic representations of images; after normalization, cosine similarity between reconstructed and ground truth images is computed to measure high-level semantic fidelity. Eff utilizes global average pooling features from EfficientNet-B1 for top-1 image classification, with the accuracy difference (1 − Acc) as a semantic distance metric reflecting deviations in the semantic decision space. The five metrics described above are implemented based on a pairwise matching protocol, wherein feature similarity calculations are performed for each reconstructed image against all real images in the validation set, thereby constructing a similarity matrix. If the reconstructed image and its corresponding real image exhibit the highest similarity in this matrix (i.e., appear in the diagonal position), it is deemed a successful match. This protocol is employed to evaluate the recognizability and semantic consistency of the reconstructed image within the high-dimensional perceptual feature space. For both real and reconstructed images, we compute their feature similarity matrix $S\in {\mathbb{R}}^{N\times N}$, where $S_{ij}$ represents the similarity score between the *i*-th reconstructed image $x_{i}$ and the *j*-th real image ${\hat{x}}_{j}$ in the perceptual feature space [53], as defined in Equation (33).

(33) $$S_{ij}=\cos(\phi (x_{i}),\phi ({\hat{x}}_{j}))=\frac{\phi (x_{i})\cdot \phi ({\hat{x}}_{j})}{\left\|\phi (x_{i})\|\cdot \|\phi ({\hat{x}}_{j})\right\|}$$

where ϕ(·) represents the feature extraction for the visual image $x_{i}$/${\hat{x}}_{j}$.

These multidimensional metrics collectively form a comprehensive evaluation framework for reconstruction performance, covering consistency from pixel accuracy to high-level semantic fidelity, thereby quantifying the model’s ability to restore images at various levels.

To validate the effectiveness of the feature extraction methods, we calculated the correlation score r_score between the extracted features and the original features *X*. Pearson’s correlation coefficient was employed to compute the correlation, as detailed in Equation (34).

(34) $$r\;=\;\frac{\sum \left(\lambda \;-\;\bar{\lambda }\right)\left(X\;-\;\bar{X}\right)}{\sqrt{\sum {\left(\lambda \;-\;\bar{\lambda }\right)}^{2}{\sum \left(X\;-\;\bar{X}\right)}^{2}}}$$

Among them, $\bar{\lambda }$ and $\bar{X}$ are the mean values of λ and X, respectively.

### 3.3. Performance Analysis and Evaluation

This section comprehensively analyzes the proposed method’s performance and mechanisms via five experiments: qualitative comparison of the reconstruction results between our method and other methods, validation of the dynamic control mechanism in the generation phase, cross-subject consistency evaluation, quantitative comparison with advanced fMRI reconstruction methods, and ablation study. Experiments were conducted on the widely used public Natural Scene Dataset (NSD) to enable thorough comparison with baseline methods and validate the proposed approach’s effectiveness.

#### 3.3.1. Qualitative Comparison of the Reconstruction Results Between Our Method and Other Methods

**Figure 7:**
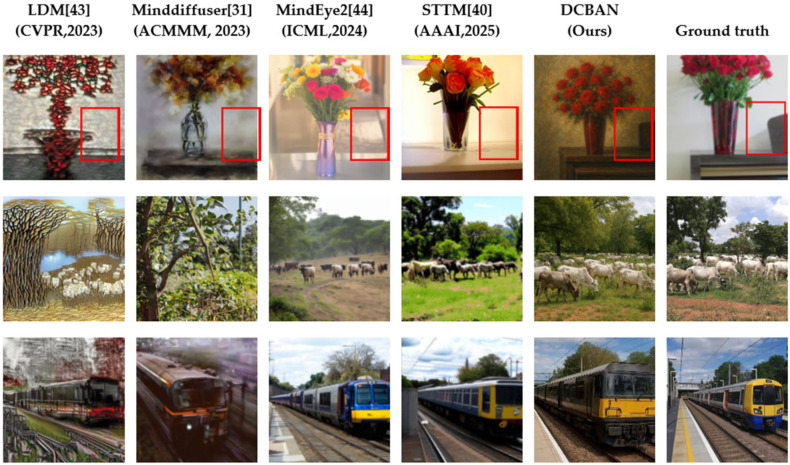
Comparison of Qualitative fMRI Reconstruction Results Between the DCBAN Model (ours) and Advanced Methods. Each row shows the visual image reconstruction of different models on the same fMRI data sample. The first four columns represent the reconstruction effects of the advanced methods Ldm [43], Minddiffuser [31], MindEye2 [44], and STTM [40], respectively. The fifth column is the reconstruction effect of our method, and the sixth column is the visual stimulus image (ground truth) corresponding to the fMRI data. Each row corresponds to one fMRI data sample.

To visually assess the generation quality of the proposed DCBAN in fMRI brain signal decoding and visual image reconstruction, the method was compared with advanced techniques such as LDM [43], Minddiffuser [31], MindEye2 [44], and STTM [40]. The comparison was performed through visual reconstruction results, as shown in Figure 7.

On the NSD, the proposed DCBAN method demonstrates exceptional reconstruction performance, capable of generating images with clear structure, rich details, and coherent semantics. Specifically, in the first row, regarding the reconstruction of the “vase” visual image, DCBAN not only accurately reconstructs the main body of the vase but also successfully reconstructs the brown background object located within the red box, a detail that none of the comparison methods were able to reproduce. The second row, concerning the “cattle herd scene” visual image, exhibits complex semantic relationships and diverse natural elements. The image reconstructed by DCBAN in this scene outperforms other methods in both semantic consistency and richness of details, with the arrangement of the cattle herd, sky, and grass being well-coordinated and natural in texture. In contrast, LDM only reconstructs a rough outline, lacking color and detail; MindDiffuser fails to correctly present the semantic structure of the cattle herd; and while MindEye2 and STTM achieve relatively close overall results, they still fall short in reconstructing the details of the grass when compared to the proposed method. In the third row, regarding the “train” visual image reconstruction, both DCBAN and MindEye2, as well as STTM, can restore rich image details. However, LDM and MindDiffuser fall short in reconstructing the complex background details, showing significant information loss.

#### 3.3.2. Validation of the Dynamic Control Mechanism in the Generation Phase

**Figure 8:**
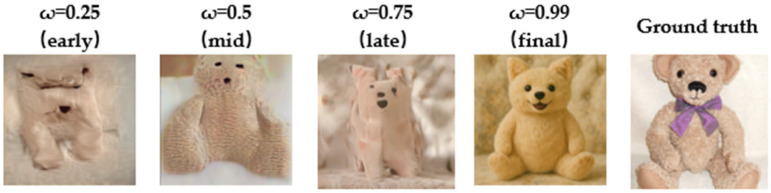
Dynamic Generation and Reconstruction Process of Visual Images by the Dynamic Confidence Adaptive Diffusion Model (DCAF) in DCBAN. Each row shows the original image and its reconstruction effects at different generation stages. Stages (early, middle, mid-late, and late) are divided based on ω = number of iterations/niter (where niter = total number of iterations). The first to fourth columns correspond to the reconstruction results of different stages in the sampling iteration process, respectively. The fifth column is the corresponding ground-truth visual image for this fMRI data sample.

To further validate the role of the Dynamic Confidence Adaptive Diffusion Model (DCAF) in the visual image generation process within DCBAN, we conducted a visual analysis of its reconstruction results at different stages. The results are shown in Figure 8. The row in the figure represents an original image and its reconstruction at different stages of the generation process. The first to fourth columns correspond to the reconstruction results at early, mid, late, and final stages of the sampling iterations. The fifth column shows the corresponding ground truth visual image. This experiment demonstrates how the DCAF mechanism strengthens semantic constraints in the early stages of image generation to stabilize the content structure, gradually releasing the generation prior as the iterations progress to enhance the richness of details and diversity of the image.

For example, “Teddy Bear” visual image reconstruction, the model begins by capturing the general location of the bear’s face and the texture of its fur in the early stages, presenting the basic structure. By the mid-stage, the overall outline becomes clearer, and by the late stage, details such as the ears emerge. In the final phase, the teddy bear’s shape, facial expression, and material details are highly restored.

#### 3.3.3. Cross-Subject Consistency Evaluation

To validate the robustness of the proposed method in addressing individual differences and its generalization ability across subjects, we conducted evaluation experiments using data from multiple subjects. The results show that the proposed DABAN model maintains stable reconstruction performance across different subjects, demonstrating its strong cross-subject generalization ability and effectiveness in mitigating the effects of individual differences in fMRI brain signals.

**Figure 9:**
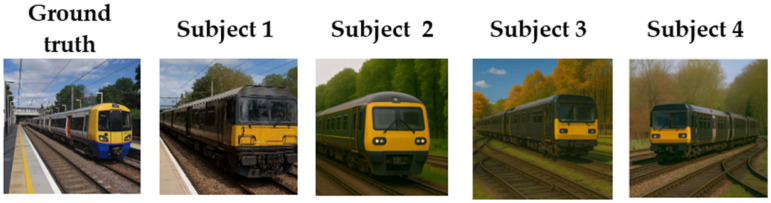
Qualitative Comparison of Reconstruction Images Obtained from Models Trained on fMRI Data Corresponding to Randomly Selected Ground-Truth Visual Stimuli. The first column shows ground-truth images (i.e., the visual image stimuli corresponding to the fMRI data). Each of the columns for Subject 1, Subject 2, Subject 3, and Subject 4 displays the reconstruction images generated from fMRI data acquired when different subjects viewed the same visual stimulus.

Figure 9 shows examples of visual image reconstruction from fMRI signals across multiple subjects. From the results, the following observations can be made. Corresponding to the “train” image, the fMRI data from all subjects successfully reconstruct a visually natural train image, with tree details at the top of the image generally matching those in the original.

#### 3.3.4. Quantitative Comparison with Advanced Reconstruction Methods

To objectively evaluate the performance of the proposed DCBAN in terms of structural fidelity, semantic consistency, and naturalness of generation, we conducted a comprehensive quantitative comparison between DCBAN and the state-of-the-art fMRI-based visual image reconstruction methods using the NSD. The specific results are presented in Table 2.

Regarding low-level perceptual metrics, DCBAN achieved the highest pixel correlation (PixCorr) of 0.361, compared to MindEyeV2’s 0.322, Minddiffuser’s 0.278, STTM’s 0.333, and LDM’s 0.246. Our method outperforms the others by 12.1%, 29.9%, 8.4%, and 46.7%, respectively. For the Structural Similarity Index (SSIM), DCBAN obtained a score of 0.423, slightly lower than MindEyeV2’s 0.431. In comparison, Minddiffuser’s SSIM was 0.354, STTM’s was 0.334, and LDM’s was 0.410. Our method improved upon these by 19.5%, 26.7%, and 3.2%, demonstrating superior structural restoration capability.

In terms of bidirectional recognition accuracy based on visual perception models, for the Alex(2) metric, MindEyeV2 achieved 96.1%, while our method reached 93.3%, which is 2.8% lower but more stable than STTM’s 95.7% and LDM’s 78.1%. This indicates that our method exhibits good generalization ability and robustness in shallow feature recognition tasks, mainly due to DeepSVD’s noise suppression and BAFRR’s adaptive fitting to different visual features. For the Alex(5) metric, our model achieved the highest value of 98.8%, a 0.2% improvement over MindEyeV2’s 98.6% and a 0.3% improvement over STTM’s 98.5%. For Inception-v3 feature recognition accuracy (Incep), our method achieved 96.0%, surpassing MindEyeV2’s 95.4% (a 0.6% improvement) and STTM’s 95.8% (a 0.2% improvement), while significantly outperforming LDM’s 83.8%. In the CLIP embedding space semantic matching (Clip), our method achieved 97.8%, an improvement of 3.3% over MindEyeV2’s 94.5% and 2.1% over STTM’s 95.7%. LDM achieved only 82.1% in this metric, demonstrating a significant improvement of 15.7% by our method. For the high-level recognition error metric (Eff), our method achieved the lowest value of 0.609, outperforming MindEyeV2’s 0.619 (approximately a 1.6% reduction), STTM’s 0.611 (approximately a 0.3% reduction), and LDM’s 0.811 (approximately a 24.9% reduction). This demonstrates that our method has stronger robustness in high-level semantic fidelity, which is largely attributed to DeepSVD’s extraction of robust features via layer-wise low-rank constraints, improving the quality of the decoded features, and to DCAF’s dynamic adjustment of condition guidance based on the reliability of the decoded features, enhancing the consistency and naturalness of the generated image details.

#### 3.3.5. Ablation Study

**Figure 10:**
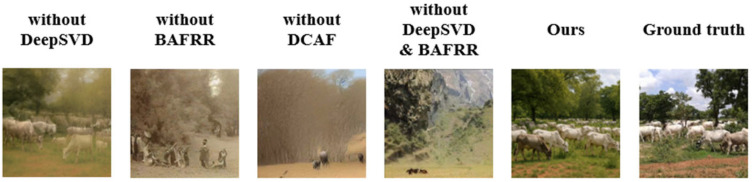
Visual Comparison of Reconstruction Results from Ablation Experiments on Each Module of DCBAN. The first five columns show the visual image reconstruction results obtained by removing the DeepSVD module, removing the BAFRR module, removing the DCAF module, removing both the DeepSVD and BAFRR modules, and using the complete algorithm (ours), with all results based on the same randomly selected single fMRI data sample. The sixth column is the corresponding visual stimulus (ground truth) for the fMRI data.

To visually and intuitively illustrate the contribution of each key module in the proposed DCBAN method to the overall performance, an ablation experiment on the reconstructed visual image was conducted using the NSD, as shown in Figure 10.

As shown in Figure 10, after removing the DeepSVD module, the details of the trees and cows became blurred, indicating the crucial role of the DeepSVD module in capturing low-level structure and detail restoration. After removing the BAFRR module, the reconstruction failed to effectively capture the semantic information of the herd of cows, instead generating an effect similar to that of a crowd, which highlights the importance of the BAFRR module in semantic reconstruction accuracy. After removing the DCAF module, the complex tree background blurred into a brown mass, and the details of the herd were reduced, demonstrating that the DCAF module plays an important role in detail reconstruction in complex scenes. After removing both the DeepSVD and BAFRR modules, although the DCAF module still preserved rich scenic details, the reconstruction lacked trees and sky, reflecting the crucial role of both the DeepSVD and BAFRR modules in feature decoding.

Meanwhile, to quantitatively evaluate the performance variation in the model on the NSD, a performance contribution analysis was conducted, and the results are presented in Table 3.

When the DeepSVD module was removed from the complete model, PixCorr dropped from 0.381 to 0.342, and SSIM decreased to 0.398, indicating the significant role of deep nested singular value decomposition in extracting stable structural features. This also had a noticeable impact on high-level semantic metrics: the Clip recognition accuracy dropped by 1.9%, Incep accuracy decreased by 1.7%, and the Eff score increased to 0.632, suggesting that the model’s semantic consistency and visual accuracy were weakened. When the BAFRR module was removed, PixCorr further decreased to 0.336, and SSIM was 0.401, showing a clear reduction compared to the full model. This indicates that the Bayesian adaptive regularization mechanism plays a crucial role in maintaining the model’s adaptive ability to different visual stimulus conditions. Correspondingly, Clip and Incep dropped by 2.2% and 2.2%, respectively, while Eff increased to 0.640, reflecting a decline in the overall quality of the decoded semantic features. After removing the DCAF module, the decline in semantic-related metrics was particularly significant: Clip accuracy dropped from 97.8% to 94.2%, Incep from 96.0% to 92.5%, while the Eff score increased to 0.651. This indicates that the absence of dynamic semantic injection control severely undermines the semantic consistency and detailed representation of the generated images. Meanwhile, both PixCorr and SSIM also decreased to 0.331 and 0.386, respectively, showing that DCAF plays a synergistic role in maintaining structural stability. When both the DeepSVD and BAFRR modules were removed, the model’s performance significantly dropped, with PixCorr at only 0.310 and SSIM at 0.375. Clip and Incep also dropped to 92.8% and 91.6%, respectively, indicating that this combination has a synergistic enhancing effect on decoding accuracy and structural feature extraction. In the weakest configuration (Baseline, removing all three modules), the model’s performance degraded across the board: PixCorr and SSIM decreased to 0.295 and 0.359, Clip dropped to 90.7%, Incep to 89.4%, and Eff rose to 0.688, demonstrating strong semantic drift and image quality degradation. This proves that the three modules are indispensable in enhancing various aspects of the model’s performance.

#### 3.3.6. Comparison of Feature Extraction Method Effectiveness

Compared to PCA, SVD, t-SNE, UMAP, CNN, NMF, and other existing methods, Principal Component Analysis (PCA) and Deep Nested Singular Value Decomposition (DeepSVD) were selected for feature extraction because their combination demonstrates significant effectiveness. This effectiveness is evaluated using the r. This metric indicates the highest correlation between the extracted features and the original features *X*, as shown in Equation (34).

As shown in Table 4, there are significant differences in the correlation coefficient r between different feature extraction methods and the original features *X*. Traditional linear methods such as PCA (r = 0.84) and SVD (r = 0.80) retain the linear structure of the original features relatively well, both achieving substantially higher correlations than nonlinear dimensionality reduction methods such as t-SNE (r = 0.41), UMAP (r = 0.48), and the deep learning model CNN (r = 0.56). This suggests that preserving linear consistency with the original features is crucial for achieving high-quality feature representations in high-dimensional data scenarios. NMF attains a moderate correlation (r = 0.67), capturing part of the structural information but still falling short of PCA and SVD. Notably, our proposed PCA + DeepSVD method, which integrates the advantages of deep nested singular value decomposition with the linear decomposition of PCA, achieves the highest correlation (r = 0.87). This indicates that the method not only inherits PCA’s strength in preserving linear structure but also further enhances consistency with the original features. Therefore, the PCA + DeepSVD combination provides the best balance between retaining original information and producing robust representations, making it the most suitable feature extraction approach.

## 4. Discussion

This study proposes the Dynamic Confidence Bayesian Adaptive Network (DCBAN), which demonstrates superior performance in fMRI-driven brain information decoding and visual image reconstruction tasks, particularly in terms of structural fidelity, model generalization capability, and image naturalness, showing significant improvements over existing methods.

In terms of visual image reconstruction quality, a cross-method comparison analysis was conducted, as shown in Figure 7. The DCBAN method proposed outperforms state-of-the-art methods, including LDM [43], Minddiffuser [32], MindEye2 [44], and STTM [39], in generating quality across several aspects, such as structural fidelity, detail restoration, and naturalness in complex scenes. To objectively assess the visual image reconstruction quality of DCBAN, quantitative comparison experiments with advanced fMRI reconstruction methods were carried out. DCBAN achieved optimal or near-optimal performance in most core metrics, particularly demonstrating a significant advantage in pixel structural fidelity and high-level semantic consistency, further confirming its leading position in fMRI decoding tasks. These advantages are primarily due to DCBAN’s incorporation of deep nested singular value decomposition (DeepSVD) in the feature extraction phase, which effectively suppresses noise in fMRI signals and enhances the model’s ability to capture structural and textural information in high-dimensional neural data. This mechanism notably strengthens the reconstruction of object edges, textures, and scene layouts, especially in complex scenes (e.g., rows 3 and 5). Additionally, in the image generation phase, DCBAN utilizes a dynamic confidence adaptive diffusion model, which dynamically adjusts the strength of semantic injection based on feature confidence, significantly improving the detail fidelity and semantic consistency of the generated images. In contrast, LDM, MindDiffuser, MindEye2, and STTM rely on static fusion strategies and fail to adaptively adjust based on feature reliability, resulting in issues such as detail loss and structural blurring during the image reconstruction process.

As shown in Figure 8, the dynamic modeling ability of conditional semantic information in the DCAF module plays an incremental role in enhancing the naturalness of reconstructed complex scenes. Compared to traditional static semantic fusion methods, DCAF introduces a confidence-aware mechanism coupled with a time decay factor for dynamic conditional modulation, enabling dual adjustments based on semantic feature reliability and the generation stage. The confidence-aware modulation dynamically weights the decoded semantic features through a lightweight confidence network (LCN), removing misleading low-reliability semantic signals and mitigating noise interference in fMRI signals. The time decay factor, as an explicit modeling parameter during the iterative stage, gradually shifts the semantic guidance strength from strong constraints to weaker ones. Early in the generation, the semantic constraint enhances structural fidelity, while later stages progressively release these semantic limitations to increase the freedom of detail and style generation, balancing global structural accuracy and local detail richness. DCAF effectively promotes the accurate restoration of image structure and controls the freedom of detail generation while maintaining decoded semantic consistency, making it particularly well-suited for handling high-dimensional noise and semantic ambiguity in fMRI brain signals.

Regarding robustness and cross-subject generalization, a cross-subject consistency evaluation experiment was performed, as shown in Figure 9. The experimental results validate the effectiveness of the DCBAN framework in cross-subject fMRI brain signal decoding tasks. Despite significant differences in neural response patterns across subjects, the model maintained high consistency in reconstructed structure, semantic coherence, and detail expression, demonstrating a strong cross-subject generalization ability. This ability is primarily attributed to the Bayesian Adaptive Feature Ridge Regression (BAFRR) in DCBAN, which dynamically adjusts the regularization level based on individual differences and the complexity of visual stimuli, thereby enhancing the model’s adaptability to diverse neural patterns. The experimental results not only validate the effectiveness of the model design but also demonstrate that DCBAN can achieve robust and accurate visual image reconstruction in real-world multi-subject brain decoding applications.

Through ablation experiments, we further verified the crucial role of each module in DCBAN, as shown in Figure 10 and Table 3. The results indicate that the DeepSVD module is essential for detail recovery and the reconstruction of low-level structures, the BAFRR module enhances the model’s adaptability, ensuring stability in the decoding process, and the DCAF module improves the detail and naturalness of the image generation phase. Removing any of these modules significantly reduces the reconstruction quality, particularly in complex scenes, where the removal of DCAF causes the generated images to lose rich details and semantic consistency. Therefore, the three key modules of DCBAN are interdependent and indispensable.

Although the proposed DCBAN model performs well in fMRI-based image reconstruction tasks, it has notable limitations. It lacks the ability to reconstruct fine details: constrained by the spatiotemporal resolution of available fMRI data, existing models (including DCBAN) are limited in capturing local image features. For instance, the “teddy bear’s” bow tie detail in Figure 8 was inaccurately reconstructed, reflecting insufficient sensitivity of the model to specific details and subtle color differences. The model adopts a single-subject training approach; while it shows high consistency in cross-subject experiments, reconstructed results still exhibit cross-subject differences in color and local texture (see Figure 9), indicating the need for further exploration in cross-subject modeling for fMRI decoding tasks. In terms of quantitative metrics in Table 3, DCBAN demonstrates significant advantages in pixel structural fidelity and high-level semantic consistency, but fails to surpass the top-performing method MindEyeV2 [44] in SSIM and Alex(2) metrics, due to the absence of data augmentation techniques to increase data diversity. Although DCBAN performs excellently on natural scene datasets, it still faces generalizability challenges when applied to other datasets or non-natural visual stimuli. Differences in imaging parameters, task paradigms, and stimulus types across datasets may lead to inconsistencies between fMRI activation patterns and the model’s training distribution, thereby reducing decoding accuracy. For non-natural stimuli such as abstract graphics, symbols, or medical images, the pre-trained visual semantic features relied on by the model cannot effectively characterize their brain response patterns, which may easily result in semantic deviation or structural distortion.

## 5. Conclusions

The DCBAN model proposed in this thesis offers a novel solution for fMRI-based visual image reconstruction, significantly enhancing the robustness and generative quality of the reconstructed images. To improve the structural fidelity of visual image reconstruction in complex fMRI decoding scenarios, DeepSVD strengthens the compact expression of features by imposing low-rank constraints on the layers of the network, learning robust low-rank features through nonlinear mappings. This effectively suppresses noise and enhances feature stability. BAFRR combines singular value space dimensionality reduction with Bayesian adaptive grid search to dynamically optimize regularization levels, improving decoding accuracy and model generalization capabilities. Additionally, DCAF introduces a confidence network to predict the reliability of semantic features, combined with a time decay mechanism to dynamically adjust semantic constraints during the diffusion generation process, thereby enhancing the diversity and naturalness of generated images in complex scenes. Experimental results on NSD demonstrate that DCBAN outperforms current state-of-the-art methods in both the quality comparison analysis and quantitative metrics (PixCorr, Alex(5), Incep, Clip, Eff) for visual image reconstruction.

Despite these advancements, our study has certain limitations, particularly regarding adaptability in dynamic perception tasks and generalization across multiple subjects and diverse scenes. Future research will focus on utilizing higher-resolution neuroimaging data to provide more potent training data for capturing complex visual details. Moreover, the introduction of cross-subject modeling and large-scale pretraining methods is expected to overcome the limitations imposed by single-subject setups, mitigating the performance bottleneck caused by individual differences and thus enhancing generalization ability at the group level. Furthermore, future research should also address data diversity, using techniques such as data augmentation to improve model performance. Its generality can be enhanced by introducing domain-adaptive learning, multi-modal pre-training, and cross-subject alignment methods. In addition, future work can also further optimize its scalability and computational efficiency through methods such as model pruning, low-rank approximation acceleration, and multi-GPU parallel training.

## Figures and Tables

**Table 1:** Hyperparameter settings and instructions (continued).

Module	Parameter Name	Type	Quantities	Reason for Selection
Deep-SVD	Nested Singular Value Decomposition Optimization Layers	Integer	57	A deeper structure captures complex spatial patterns; 57 strikes the optimal balance between performance and computational cost.
	Initial Learning Rate	Continuous	10^−3^	A larger learning rate benefits early-stage fast convergence, paired with learning rate decay for stable optimization.
	Singular Value Decomposition Objective Function Parameters $\beta_{1}$, $\beta_{2}$, $\beta_{3}$	Continuous	$\beta_{1}$ = 1, $\beta_{2}$ = −1, $\beta_{3}$ = 1	Controls the positive-negative balance of decomposition constraints. Preliminary experiments show this combination achieves the best reconstruction accuracy.
	Adam Optimizer Parameter $\beta_{1}$, $\beta_{2}$, ε	Continuous	$\beta_{1}$ = 0.9, $\beta_{2}$ = 0.999, ε = 10^−8^	Standard stable configuration for adapting to sparse, high-dimensional fMRI feature training.
BAFRR	SMALL_BIAS	Continuous	10^−2^	Controls small bias terms in grid initialization to ensure numerical stability.
	BIG_BIAS	Continuous	10^−3^	Sets a larger bias range to cover the possible solution space.
	Log Space Sampling Step Size A	Continuous	0.2	Balances search accuracy and computational efficiency.
	Kernel Length Scale Initial Value	Continuous	1	Matches amplitude, maintaining initial scale invariance.
	Non-uniform Adaptive Grid Redistribution s	Continuous	1.75	Controls the rate of change in grid sparsity density.
	Focus Adjustment Parameter c	Continuous	3	Enhances fitting accuracy in high-gradient regions.
	Bayesian Optimization Iterations	Integer	20	Balances search space exploration and time cost.
	Initial Regularization Search Range	Interval	[0, 1]	Covers the most common range of regularization strengths.
	Target Score Grid Step Size	Continuous	0.05	Ensures search accuracy while reducing computational load.
	Cross-validation Folds k	Integer	5	Improves evaluation stability with limited samples.
DCAF	Confidence Network Bottleneck Dimension	Integer	4	Reduces feature dimension to prevent overfitting while retaining key information.
	Time Decay Step Range	Interval	[0, 50]	Controls the confidence decay rate, balancing short-term and long-term dependencies.
	Diffusion Sampling Steps	Integer	50	Balances generation quality and inference time.
	DDIM Step Size Parameter η	Continuous	0.0	Ensures experimental reproducibility.
General Settings	Random Seed	Integer	42	Ensures experimental reproducibility.
	Early Stopping Strategy	Boolean	Enabled	Prevents overfitting and saves computational resources.

**Table 2:** Comparison of Quantitative Metrics Between DCBAN and Advanced fMRI Visual Image Reconstruction Methods on the NSD. The first four rows present the quantitative metrics of the reconstruction results from advanced methods, and the fifth row shows those of our method. Each column represents a different evaluation metric. The best results are emphasized in bold.

Method	PixCorr↑	SSIM ↑	Alex(2) ↑	Alex(5) ↑	Incep ↑	Clip ↑	Eff ↓
MindEyeV2 [44]	0.322	**0.431**	**96.1%**	98.6%	95.4%	94.5%	0.619
Minddiffuser [31]	0.278	0.354	-	-	-	76.5%	-
STTM [40]	0.333	0.334	95.7%	98.5%	95.8%	95.7%	0.611
LDM [43]	0.246	0.410	78.1%	85.6%	83.8%	82.1%	0.811
**Ours**	**0.361**	0.423	93.3%	**98.8%**	**96.0%**	**97.8%**	**0.609**

**Table 3:** Comparison of Quantitative Metrics for Visual Image Reconstruction from Ablation Experiments on Each Module of DCBAN. Each row represents the evaluation of metrics for visual image reconstruction results obtained by removing the DeepSVD module, removing the BAFRR module, removing the DCAF module, removing both the DeepSVD and BAFRR modules, removing all modules, and using our proposed complete algorithm, with all experiments conducted on fMRI data samples. Each column represents a different evaluation metric.

Method	PixCorr ↑	SSIM ↑	Incep ↑	Clip ↑	Eff ↓
without DeepSVD	0.342	0.398	94.3%	95.9%	0.632
without BAFRR	0.336	0.401	93.8%	95.6%	0.640
without DCAF	0.331	0.386	92.5%	94.2%	0.651
without DeepSVD and BAFRR	0.310	0.375	91.6%	92.8%	0.667
Baseline (No three modules)	0.295	0.359	89.4%	90.7%	0.688
Ours	0.381	0.293	96.0%	97.8%	0.609

**Table 4:** Quantitative Comparison Between Our Combined Method of PCA and DeepSVD and Other Feature Extraction Methods. The first six columns, respectively, show the correlation index r between the features extracted by PCA, SVD, t-SNE, UMAP, CNN, NMF and the original feature *X*. The last column shows the correlation index r between the features extracted by our method and the original feature *X*.

PCA	SVD	t-SNE	UMAP	CNN	NMF	PCA + DeepSVD (Ours)
0.84	0.80	0.41	0.48	0.56	0.67	0.87

## Data Availability

Our source code will available at https://github.com/caiyy1023/DCBAN-A-Dynamic-Confidence-Bayesian-Adaptive-Network (accessed on 9 July 2025). The fMRI dataset [45] used in this research were obtained from public domain and are available online at https://naturalscenesdataset.org/ (accessed on 9 July 2025).
